# Retraction: Gastroprotection studies of Schiff base zinc (II) derivative complex against acute superficial hemorrhagic mucosal lesions in rats

**DOI:** 10.1371/journal.pone.0341791

**Published:** 2026-01-28

**Authors:** The PLOS One Editors

After this article [1] was published, concerns were raised regarding results presented in Figs 3 and 4.

Specifically:

- Fig 3B of this article [1] appears to partially overlap with Fig 2q of [2], and Fig 7E of [3], despite representing different experimental conditions.
- In Fig 4, the following lanes appear more similar than would be expected from independent results:
  - Hsp70 lanes 1 and 2
  - Hsp70 lane 3, Bax lane 4, and Bax lane 6
  - Hsp70 lanes 5 and 6
  - Bax lanes 1 and 2

The authors did not comment on the above concerns.

In light of the above concerns that question the reliability and integrity of the reported results, the *PLOS One* Editors retract this article.

NAM agreed with the retraction. SG, NSG, PH, MH, BK, MAA, HMA, and AHAH either did not respond directly or could not be reached.
